# Th17/Treg imbalance is associated with reduced indoleamine 2,3 dioxygenase activity in childhood allergic asthma

**DOI:** 10.1186/s13223-020-00457-7

**Published:** 2020-07-07

**Authors:** Ying Hu, Zhiqiang Chen, Jing Zeng, Shouyan Zheng, Liujuan Sun, Li Zhu, Wei Liao

**Affiliations:** Department of Pediatrics, The First Affiliated Hospital of Army Medical University, Chongqing, 400038 China

**Keywords:** Th17/Treg imbalance, IDO, Childhood allergic asthma

## Abstract

**Background:**

The differentiation of CD4+ lymphocytes Th17/regulatory T cells (Treg) and indoleamine 2,3-dioxygenase (IDO) is associated with the pathogenesis of allergic asthma. Basic research has shown that IDO is likely a “switch” of the transition from Th17 cells to Tregs under certain conditions. However, no relevant clinical studies have been reported on the association between IDO activity and Th17/Treg imbalance in children with allergic asthma. The goal of this study was to test whether indoleamine 2,3 dioxygenase (IDO) participates in the pathogenesis of pediatric allergic asthma by influencing Th17/regulatory T cell (Treg) differentiation and related cytokines.

**Methods:**

Thirty-three children with allergic asthma and 33 healthy children were selected. The subjects were evaluated via a pulmonary function test, a skin prick test, and an eosinophil count. Peripheral blood was collected to measure Th17/Treg percentages and related cytokine levels. Blood and induced sputum were obtained to measure the IDO level.

**Results:**

Compared with the control group, the patient group had an obvious Th17/Treg imbalance; their IDO levels were significantly lower, their IL-17 and IL-6 levels were markedly higher, and their IL-10 and TGF-β levels were markedly lower than those of the control group. The IDO levels in both blood and induced sputum were negatively correlated with the Th17/Treg ratio.

**Conclusions:**

A significant correlation was observed between IDO activity and Th17/Treg imbalance in children with allergic asthma. IDO may upregulate Treg numbers by stimulating IL-10 production and inhibiting IL-6 expression. Therefore, IDO may be a molecular switch that leads to the conversion of Th17 cells to Tregs, thus playing a potentially protective role in the pathogenesis of asthma.

*Trial registration* This study was approved by the Chinese Clinical Trial Registry with registration number ChiCTR-COC-15006080 and was reviewed and approved by the Ethics Committee of Southwest Hospital. The name of registration: The effect of indoleamine 2,3 dioxygenase (IDO) on Regulation of Th17/Treg Differentiation in Childhood Asthma. Date of registration: 14/03/2015. URL of trial registry record: http://www.chictr.org.cn

## Background

Allergic asthma (asthma) is an allergic inflammatory disease characterized by airway hyperresponsiveness. The Th1/Th2 imbalance is key in the pathogenesis of asthma. In recent years, Th17 cells and regulatory T cells (Tregs) have also been found to play an important role in the pathogenesis of asthma. Studies in animal models have demonstrated that the ability of Tregs to inhibit the inflammatory response of Th2 cells is key to the development of tolerance to asthma. Th17 cells, a subpopulation of CD4+ T cells first discovered in 2005, are characterized by the secretion of IL-17. In addition to having a strong relationship with eosinophilic airway inflammation in asthma, IL-17 mediates airway neutrophilic inflammation in asthma and is correlated with the severity of asthma [1, 2]. Zhao et al. [3] demonstrated in a chronic airway inflammation model that Th17 cells inhibit Treg-mediated tolerance and concluded that chronic airway inflammation could be improved through regulation of the Th17/Treg axis.

Dendritic cells (DCs) are the most effective antigen-presenting cells (APCs) in the airway. In addition to presenting antigens and inducing immune responses and asthma, airway DCs also induce CD4+ T cells to differentiate into Tregs and thus induce immune tolerance, preventing the onset of an asthma episode. Plasmacytoid DCs (pDCs) in the airway can induce immune tolerance. Therefore, the shift of airway CD4+ T cells to effector T cells or Tregs depends on the different DC subpopulations that present the antigen. Some researchers have even suggested that respiratory immune tolerance may be a function of the subtype of pDCs [4]. Interestingly, Hayashi et al. [5] found that the tolerogenic activity of airway pDCs is achieved through the expression of indoleamine 2,3 dioxygenase (IDO), which inhibits T cell differentiation.

IDO is the rate-limiting enzyme for the catabolism of tryptophan and plays an important role in peripheral tolerance. In addition to its roles in immune tolerance in pregnancy, organ transplantation and cancer, IDO was recently found to have key functions in allergic diseases [6, 7]. Raitala et al. [8] demonstrated that IDO activity was lower in allergy patients than in healthy individuals. Among allergy patients who were exposed to airborne allergens, those without clinical symptoms exhibited higher plasma IDO activity than patients with symptoms and healthy individuals [9]. Additionally, patients with mild intermittent or mild-to-moderate persistent asthma had lower baseline IDO activity in induced sputum when compared with healthy individuals. IDO activity increased markedly in response to glucocorticoid inhalation, to a certain extent through stimulating IL-10 production [10]. Another report showed that simvastatin enhanced the glucocorticoid-mediated regulation of the Th17/Treg imbalance by upregulating IDO and IL-10 expression and downregulating IL-6 and IL-23 expression [11]. Similarly, tests with induced sputum from asthma patients showed that statins enhanced the anti-inflammatory effects of glucocorticoids by upregulating IDO activity and IL-10 [12]. Adipose-derived stem cells alleviated allergic airway inflammation and improved lung functions in asthmatic patients through Treg amplification, and the induction of Tregs involved the secretion of soluble factors such as IDO and TGF-β, the downregulation of Th2 cytokines and the upregulation of Th1 cytokines (IFN-γ) and regulatory cytokines (IL-10 and TGF-β) [13]. Furthermore, previous studies have often examined the prevalence and pathogenic roles of the Th17/Treg balance in children with allergic asthma or rhinitis alone [14, 15]. Therefore, we speculated that the Th17/Treg imbalance is associated with IDO in children with allergic asthma and that the underlying mechanisms may be related to regulation of the corresponding cytokines. At present, no clinical studies have reported on the relationship between IDO and the Th17/Treg imbalance in relation to pediatric allergic asthma. This study investigated the association between IDO activity and Th17/Treg expression and the possible molecular mechanisms in pediatric patients older than 5 years with allergic asthma compared with children without any allergic symptoms.

## Methods

The datasets generated and/or analyzed during the current study are available from the corresponding author upon reasonable request.

This study was approved by the Chinese Clinical Trial Registry with registration number ChiCTR-COC-15006080 and was reviewed and approved by the Ethics Committee of The First Affiliated Hospital of Army Medical University. The methods were carried out in accordance with the approved guidelines. The study was conducted in compliance with the principles of the Declaration of Helsinki. Written informed consent was obtained from the parents of all enrolled subjects. The authors have access to information that could identify individual participants during or after data collection.

### Subjects

The subjects were recruited at the Pediatric Outpatient Department of Southwest Hospital. A total of 30 (the number of cases was estimated based on the significance of the expected difference) pediatric patients with allergic asthma (according to retrospective grading of disease severity) older than 5 years were enrolled, along with 30 age- and sex-matched healthy children who underwent a physical examination during the same period (control group). The average age and sex distributions of the patients were considered in the selection of subjects for each group to reduce deviations and ensure a normal distribution as much as possible. The clinical data of the 2 groups are comparable.

The sample size was calculated using the superiority test formula for a two-sample mean comparison based on a two-group parallel controlled randomized block design:

$$ N = \frac{{\left( {Z_{1 - \alpha } + Z_{1 - \beta } } \right)\left( {Z_{1 - \alpha } + Z_{1 - \beta } } \right)^{2} \times \left( {\sigma_{1}^{2} + \sigma_{2}^{2} } \right)^{2} }}{{\left( {\mu_{1} - \mu_{2} - \delta } \right)^{2} }} $$

The observation indicator was IDO activity. According to relevant studies in China and other countries, the characteristic value of IDO activity, which is the ratio of kynurenine to tryptophan content (unit: %), was 12.8 in the experimental group and 7 ± 1 in the control group. Thus, α = 0.025 (unilateral), 1 – β = 0.90, *μ*_1_ = 12.8, *μ*_2_ = 7, $\sigma_{1}^{2} = 2^{2}$, $\sigma_{2}^{2} = 1^{2}$, and δ = 3. The sample size of each group was calculated to be 29. Considering interference factors such as subject dropout during the clinical trial, we determined that each group should have 30 subjects.

### Inclusion criteria for the observation group

The inclusion criteria for the observation group were as follows: (1) patients aged 5 years or older; (2) patients who were diagnosed with asthma for the first time or were previously diagnosed with asthma but had not been treated with standard asthma therapies; and (3) patients who had not received treatment with an inhaled glucocorticoid or leukotriene receptor antagonist within 2 weeks prior to the study.

### Diagnosis criteria for asthma

1. Recurrent wheezing, coughing, shortness of breath and chest tightness that frequently occurs or is aggravated at night and (or) in the early morning and is mostly caused by exposure to allergens, cold air, physical and chemical stimuli, respiratory infection and physical activity;
2. Audible scattered or diffuse wheezes mainly during the expiratory phase in both lungs during an attack, with a prolonged expiratory phase;
3. The ability for the above symptoms and signs to be effectively relieved by asthma treatment or to be self-resolved;
4. Exclusion of other diseases as the cause of wheezing, coughing, shortness of breath or chest tightness;
5. Patients with atypical clinical manifestations (such as no obvious panting or wheezing) should have at least one of the following: (1) positive results on a bronchial provocation test or exercise challenge test; (2) confirmed existence of reversible air flow restriction, with positive results on a bronchial diastolic test, namely, a greater than 12% increase in forced expiratory volume during the first second (FEV1) 15 min after inhaling the fast-acting β2 receptor agonist salbutamol sulfate; and (3) daily variability in peak expiratory flow (PEF) (continuous monitoring for 1–2 weeks) of 20%.

Patients who met items (1) to (4) (12 subjects) or items (4) and (5) (18 subjects) were diagnosed with asthma.

### Inclusion criteria for the control group

A total of 30 healthy children (with no asthma, recent infectious disease, autoimmune disease or cardiopulmonary disease and no treatment with glucocorticoids or immunomodulators within 2 weeks) who underwent a physical examination at the outpatient office of the Department of Pediatrics were recruited during the same time period and had the desired age and sex distribution.

### Exclusion criteria

1. Patients who were given oral or intravenous glucocorticoids or immunomodulators within 2 weeks prior to the study;
2. Patients who had complications of other immune disorders, such as tuberculosis, systemic lupus erythematosus and juvenile idiopathic arthritis;
3. Patients who had infectious diseases, such as an upper respiratory tract infection and pneumonia, within 4 weeks of the onset of the asthma episode;
4. Patients who had cardiopulmonary failure.

### Study design

Part I: Collection of complete medical history, physical examination, pulmonary function tests, skin prick test for allergens and eosinophil count (EC).

Pulmonary function tests:

Quality control standard of children’s lung function: Exhale rapidly to PEF, no hesitation, smooth breath descending branch, no cough and glottis closing; Platform to achieve end expiratory flow; If flow at the end > 10%, It can be considered as early termination; deally, at least two acceptable tests should be completed, and the difference between two FVC and FEVT should be less than 0.1 L or 10%.

2. Skin prick test for allergens: allergenicity was defined as a positive skin reaction to more than one of the 12 common inhalant allergens, such as 2 types of dust mites, pollen, cat hair, dog hair, *Alternaria* and *Aspergillus*.

Implementation steps: Drop a drop of allergen test solution onto the sterilized skin on the palmar side of the forearm of the subject, and then prick it vertically with a point prick. The negative and positive control groups were set with normal saline and histamine diphosphate solution. According to the papule area ratio caused by pricking solution and positive control, the reaction level was judged: > 25% was positive, < 25% was negative.

Part II: Peripheral blood tests.

After enrollment, blood samples were immediately collected from the subjects in the observation group and the control group, and the samples were processed as follows:

1. *Specimen collection:* Four milliliters of venous blood (with heparin as an anticoagulant) was collected from the subjects. Whole blood was centrifuged, and plasma was collected and stored at − 80 °C. Peripheral blood mononuclear cells (PBMCs) were isolated via density gradient centrifugation in Ficoll.
2. *Tested parameters*

1) Flow cytometry was used to detect Th17 (CD4+ IL17+) cells and Tregs (CD4+CD25+Foxp3+).

Antibodies used and isotype controls:

- Human IL–17/IL–17A PE–conjugated Antibody (IC317P); Mouse IgG2B PE-conjugated
- Isotype control (IC0041P).
- Human/Mouse FoxP3 PE– conjugated Antibody (IC8214P-100); Normal Rabbit IgG.
- PE-conjugated control (IC105P)
- Human CD25/IL-2 R alpha APC– conjugated Antibody (FAB1020A-100); Mouse IgG2A APC-conjugated Antibody (IC003A)
- Human CD4 Fluorescein –conjugated Antibody (FAB3791F-100); Mouse IgG2A Fluorescein-conjugated Antibody(IC003F) gating procedures: Th17, the gate was set with CD4, and the proportion of CD4+ IL17+ in the gate was analyzed. Treg set the gate with CD4 and SSc. CD4 cells were selected to analyze the expression of CD25+ and Foxp3+, and the proportion of Foxp3 cells was analyzed.

Cell fixation and or freezing employed:

- FlowX FoxP3/Transcription Factor Fixation & Perm Buffer Kit (FC012-100)
- Flow Cytometry Staining Buffer (1×)(FC001).

Machine and software involved:

BD Accuri™ C6 software is Flowjo 7.6.1 Min

2) Enzyme-linked immunosorbent assay (ELISA) was utilized to examine the levels of cytokines, including IL-10, IL-17, IL-6 and TGF-β, in plasma.

Human TGF-beta 1 Quantikine ELISA Kit (DB100B),Human IL-17

Quantikine ELISA Kit (D1700), Human IL-6 Quantikine ELISA Kit (D6050)

Human IL-10 Quantikine ELISA Kit (D1000B) were used. 3) High-performance liquid chromatography (HPLC) was employed to determine tryptophan and kynurenine concentration. IDO activity was calculated as kynurenine concentration (μmol/L)/tryptophan concentration (μmol/L).

It employed a Symmetry Shield RP-C18 column (150 mm × 3.9 mm i.d., 5 μm) and a mobile phase of 15 mmol/L sodium acetate-acetic acid solution containing 2.7% (v/v) acetonitrile (pH 3.6) at a flow rate of 1.0 mL/min. The ultraviolet detector was Operated at 225 nm. Serum samples were first precipitated with a 5.0% perchloric acid solution, then centrifuged to remove protein residue and finally analyzed by HPLC.

Part III: Induced sputum test

1. *Specimen collection:* After enrollment, induced sputum samples were immediately collected from the subjects in the observation and control groups. Sputum was aspirated after subjects were given aerosolized 3% hypertonic saline (1.5 mL of 10% NaCl + 2.5 mL of 0.9% NaCl), and the induced sputum supernatant was stored at − 70 °C.
2. *Tested parameters:* HPLC was employed to determine tryptophan and kynurenine concentration. IDO activity was calculated as kynurenine concentration (μmol/L)/tryptophan concentration (μmol/L).

### Statistical methods

All data were statistically analyzed using the software SPSS18.0. Normality tests (Kolmogorov–Smirnov and Shapiro–Wilk tests) and a test for the homogeneity of variance (Levene test) were performed before multigroup comparisons. An independent samples t test was used for intergroup comparisons of data with a normal distribution and homogeneity of variance, and the experimental results are presented as the mean (95% confidence interval). The Mann–Whitney U test for nonparametric statistical analysis was used for data that did not have a normal distribution, and the experimental results are presented as the median (quartile). Multivariate linear regression analysis was used to analyze correlations among multiple groups, and differences of P < 0.05 were considered statistically significant.

## Results

### Clinical and laboratory characteristics of the allergic asthma and healthy control groups

A total of 30 patients (5–13 years old, 17 males and 13 females) were included in the observation group of this study, with 30 age- and sex-matched healthy children in the control group. The clinical characteristics of the subjects are summarized in Table 1. Consistent with the expectations, the age and sex of the 2 groups were matched, with no statistically significant differences. Compared with the healthy control children, the children with allergic asthma had a significantly higher positive rate on the skin prick test (P < 0.001) (allergenicity was defined as a positive skin reaction to more than one of the 12 common inhalant allergens, such as 2 types of dust mites, pollen, cat hair, dog hair, *Alternaria* and *Aspergillus*). Additionally, the eosinophil count was significantly higher (P < 0.001), and the pulmonary function indicator FEV1.0/FVC% was markedly lower (P < 0.001) in the children with allergic asthma.

**Table 1:** Clinical and laboratory characteristics of subjects in two group

Variable	Patients (n = 30)	Controls (n = 30)	P value
Age, median (IQR), years	7.5 (5–9.25)	7 (5.75–11)	0.77
Sex, male/female	17/13	14/16	0.606
Allergen (positive/negative)	21/9	6/24	< 0.001
Eosinophils in blood, average (95% CI)	2.97 (2.662–3.285)	2.207 (2.032–2.381)	< 0.001
FEV1.0/FVC%	75.3 ± 12.03	91.2% ± 8.76	< 0.001
IDO in sputum, average (95% CI)	5.442 (4.950–5.935)	8.912 (8.398–9.426)	< 0.001
IDO in blood, average (95% CI)	3.136 (2.787–3.485)	4.12 (3.638–4.603)	0.001
Treg, average ± SD (%)	3.97 ± 2.052	5.69 ± 2.434	< 0.01
Th17/Treg, average ± SD	0.45 ± 0.430	1.04 ± 0.666	0.002

IDO unit: %; Eosinophils unit: %

*IDO* indoleamine 2,3-dioxygenase, *FEV1.0/FVC%* maximum expiratory rate in the first second, *IQR* interquartile range, *CI* confidence interval

### IDO activity in induced sputum and plasma in the allergic asthma and control group

**Fig. 1:**
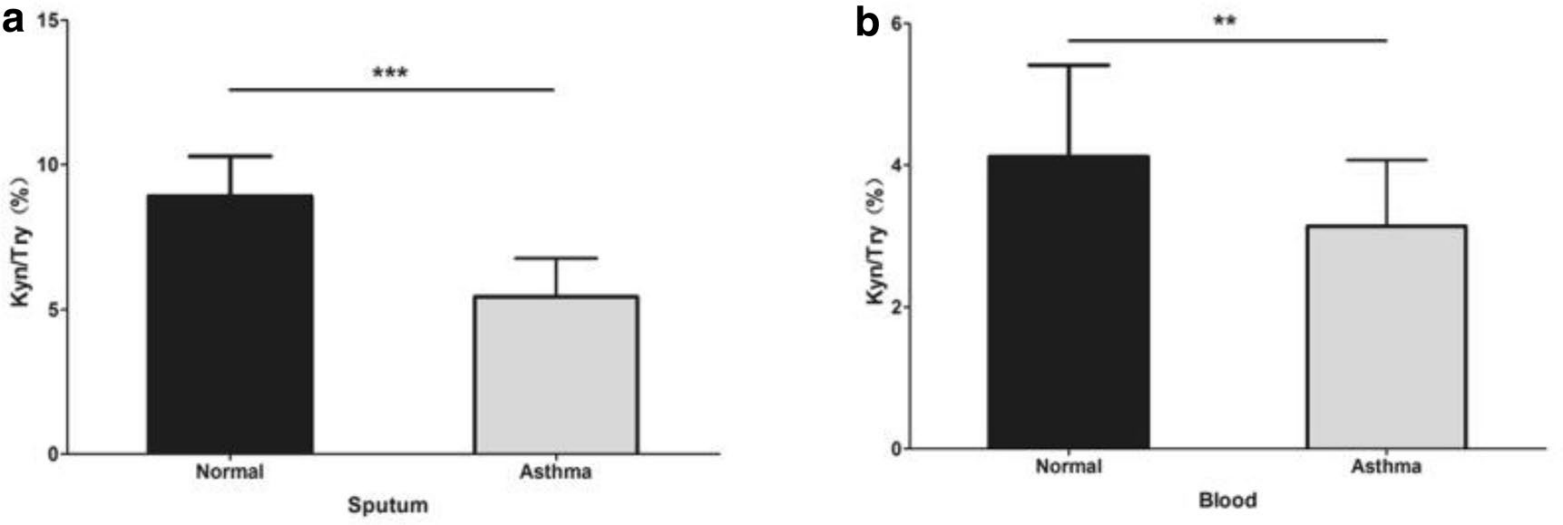
**a** Bar plot showing IDO activity in the induced sputum of subjects in two group. (* P < 0.05; ** P < 0.01; *** P < 0.001). **b.** Bar plot showing IDO activity in the blood of subjects with or without allergic asthma (*P < 0.05; **P < 0.01; ***P < 0.001)

The IDO activity was significantly lower in the pediatric patients with allergic asthma than in the control patients in both induced sputum (P < 0.001, Table 1, Fig. 1a) and peripheral blood (P = 0.001, Table 1, Fig. 1b).

### Th17 and Treg populations in the peripheral blood of the allergic asthma and control group

**Fig. 2:**
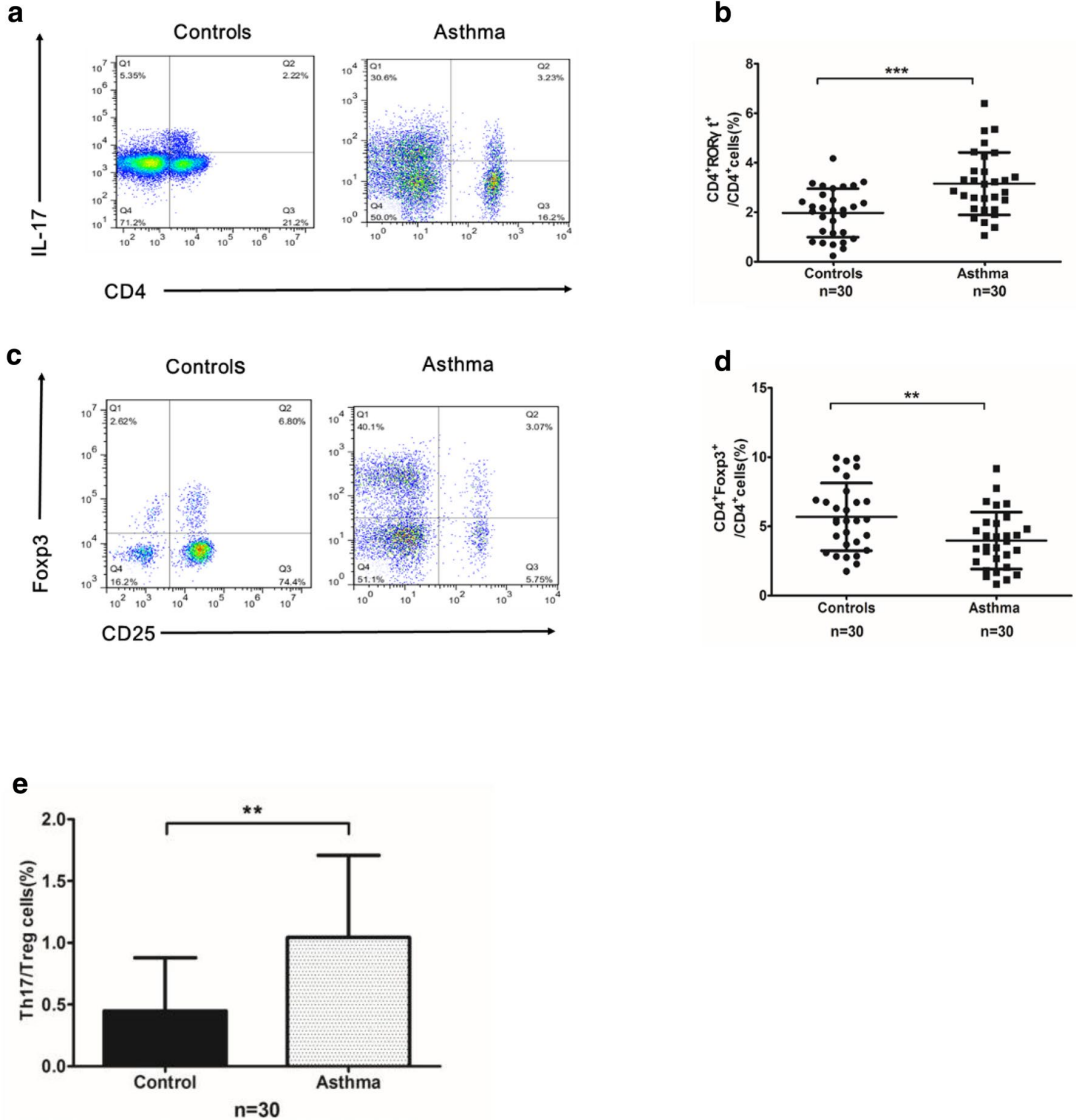
**a** FACS data for Th17 cells (CD4+ RORγt+ IL17+) in the blood of subjects in two group. **b** Scatter plot of Th17 cell data for the blood of subjects in two group. **c** FACS data for Tregs (CD4+ CD25+ Foxp3+) in subjects with or without allergic asthma. **d** Scatter plot of Treg data for the blood of subjects with or without allergic asthma. **e** Bar plot of the Th17/Treg ratio in the blood of subjects in two group (**P < 0.01)

The populations of Th17 cells (CD4+ RORγt+ IL17z+) and Tregs (CD4+ CD25+ Foxp3+) among peripheral PBMCs were examined using flow cytometry for both the allergic asthma and healthy control groups (Fig. 2a, c). The Th17 cell population was significantly higher in the asthma group than in the healthy control group (Fig. 2b), while the Treg population was significantly lower in the asthma group than in the healthy control group (Fig. 2d). Moreover, compared to the healthy control group, the asthma group showed a remarkably elevated Th17/Treg ratio (Fig. 2e).

### Expression levels of cytokines related to Th17 and Tregs in peripheral blood

**Fig. 3:**
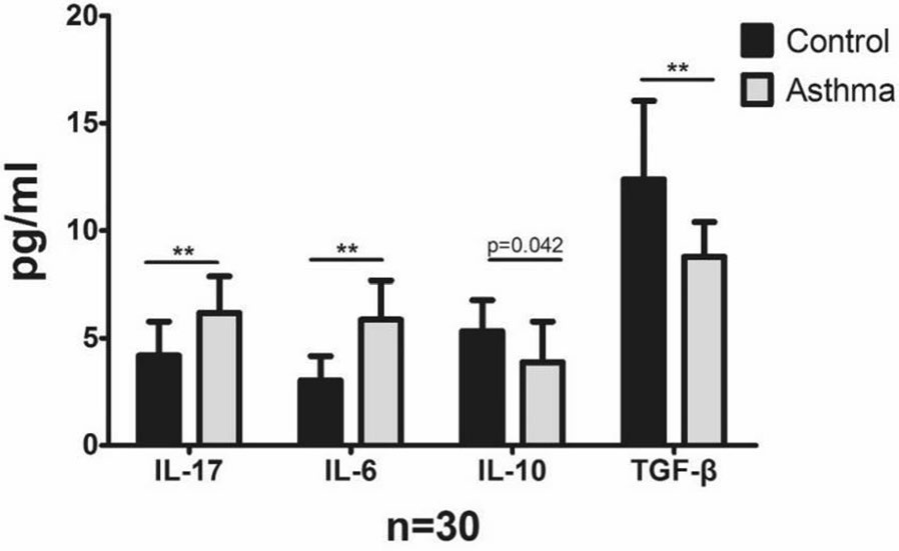
Bar plot showing expression levels of cytokines related to Th17 and Tregs in peripheral blood in two groups

The plasma levels of cytokines related to Th17 cells and Tregs, including IL-17, IL-6, IL-10 and TGF-β, were examined (Table 2, Fig. 3). IL-17 and IL-6 levels were significantly higher in plasma of children with allergic asthma than in those of the healthy control children (P < 0.01, P < 0.01), while TGF-β and IL-10 expression levels were markedly lower in the allergic asthma group than in the healthy control group (P < 0.01).

**Table 2:** Expression of Th17 and Treg effectors in the blood of subjects in two group

	Asthma (n = 30) (pg/mL)	Control (n = 30) (pg/mL)	P value
IL-17	6.18 ± 1.68	4.19 ± 1.58	< 0.01
IL-6	5.86 ± 1.80	3.03 ± 1.13	< 0.01
IL-10	3.88 ± 1.89	5.32 ± 1.45	0.042
TGF-ß	8.77 ± 1.62	12.38 ± 3.66	< 0.01

### Correlative analysis of IDO activity in induced sputum and peripheral blood and the Th17/Treg imbalance in pediatric patients with allergic asthma

**Fig. 4:**
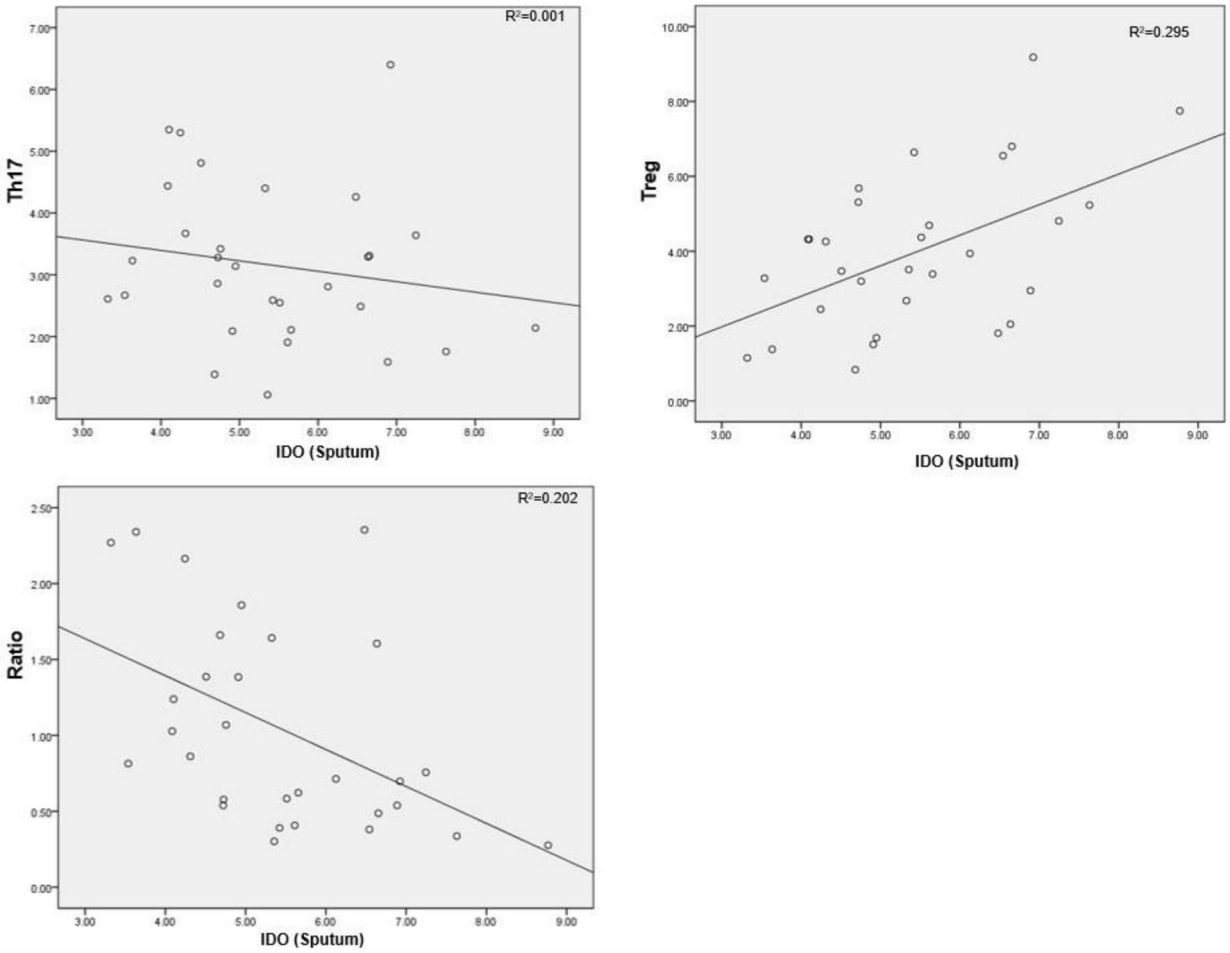
Relationship between IDO and Th17 cells, Tregs in the induced sputum of subjects with allergic asthma

**Fig. 5:**
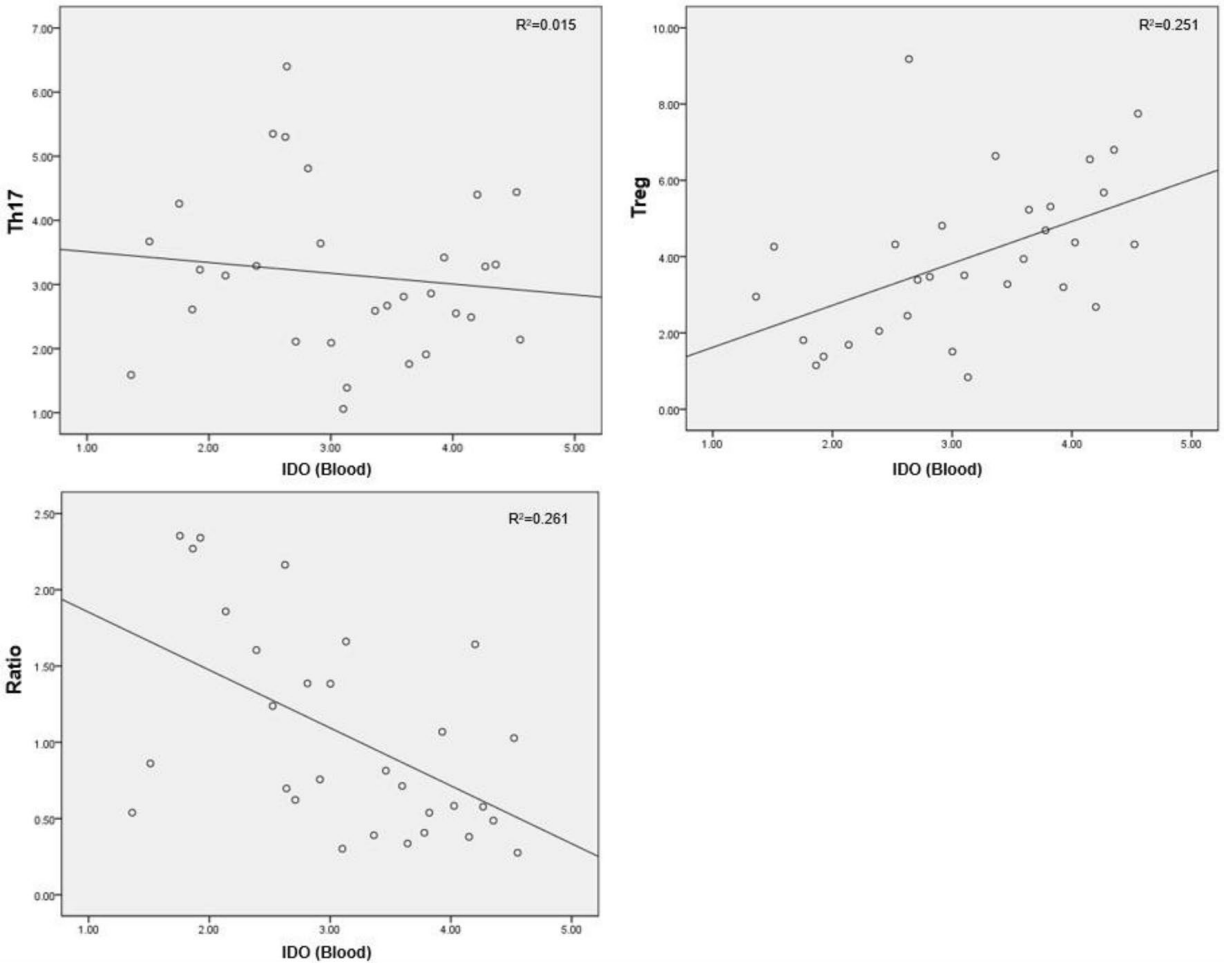
Relationship between IDO and Th17 cells, Tregs in the blood of subjects with allergic asthma

In children with allergic asthma, the IDO concentration in the induced sputum was positively correlated with the peripheral concentration of Tregs, was not significantly correlated with the Th17 cell concentration and was negatively correlated with the Th17/Treg ratio (Table 3, Fig. 4). The peripheral IDO concentration exhibited the same relationships with Th17, Treg and Th17/Treg (Table 4, Fig. 5).

**Table 3:** Relationship between IDO and Th17 cells, Tregs in the induced sputum of subjects with allergic asthma

IDO (sputum)	Th17	Treg	Th17/Treg
Pearson correlation	− 0.176	0.524	− 0.418
P value (two-sided)	0.352	0.003	0.007

**Table 4:** Relationship between IDO and Th17 cells, Tregs in the blood of subjects with allergic asthma

IDO (blood)	Th17	Treg	Th17/Treg
Pearson correlation	− 0.124	0.501	− 0.532
P value (two-sided)	0.514	0.005	0.002

## Discussion

Th1/Th2 imbalance is key in the pathogenesis of allergic asthma, with Th2 cells playing a particularly important role. However, in recent years, Th17 and Tregs have been found to have a critical function in asthma pathogenesis. Tregs are different from Th1 and Th2 cells; they have immunosuppressive activities and are primarily regulated by FOXP3. CD4+ CD25+ Tregs are currently considered to play a pivotal role in peripheral tolerance, and therefore, Treg generation is important in the airway immune tolerance of allergens [16]. Allergen-specific immunotherapy (allergen-SIT) has been shown to be effective in the prevention and treatment of asthma due to the production of inducible Tregs (iTregs) induced by repeated stimulation by allergens. Airway Treg-induced immune tolerance is likely related to the expression of cytotoxic T lymphocyte-associated antigen 4 (CTLA-4) and the secretion of cytokines such as IL-10 and TGF-β by Tregs [17]. Therefore, in children with allergic asthma, Treg populations are suppressed, and the expression of cytokines such as IL-10 and TGF-β are also significantly reduced. Th17 cells, a new CD4+ T cell subpopulation discovered in 2005, are characterized by the secretion of IL-17. The orphan nuclear receptor RORγt is an important transcription factor in Th17 cells. IL-17 not only mediates neutrophilic airway inflammation but also regulates eosinophilic and macrophagic inflammation and is thus closely associated with the pathogenesis of asthma [18]. Th17 cells and Tregs are derived from common naïve T cells and differentiate in response to different cytokines. Th17 cells and Tregs mutually suppress the differentiation of each other and play an important role in maintaining immune homeostasis. Therefore, during an asthma attack, CD4+ T cells shift to both Th2 and Th17 cells under the influence of certain factors and inhibit the formation of Tregs. This study showed an imbalance in the Th17/Treg ratio in pediatric patients with allergic asthma aged over 5 years. Compared to healthy children of the same age, the allergic asthma patients exhibited significantly increased IL-17 and IL-6 expression levels and a marked decline in TGF-β and IL-10 expression levels.

The immune regulation and induction by IDO in infections, pregnancy, and autoimmune and tumor-like lesions have been widely recognized. IDO has been detected in eosinophils, endothelial cells and pulmonary epithelial cells, indicating its role in allergic reactions [19, 20]. At present, IDO-mediated peripheral tolerance is believed to be possibly related to the following factors: (1) the inhibition of T cell differentiation and proliferation through tryptophan catabolism in the local microenvironment by IDO and the induction of T cell apoptosis by metabolites produced by IDO degradation; and (2) IDO-mediated binding of ligands on pDCs, such as TLR9, CTLA4, CD200 and GITR, to the corresponding receptors on T cells to induce the formation of antigen-specific Tregs, which stimulate pDCs to express IDO and thus inhibit T cell activation through reverse signaling (the IKKα-mediated nonclassical NF-κB signaling pathway) [21]. However, there are no published reports on IDO activity in the induced sputum in the airways of children with allergic asthma in China or other countries. This study found significantly reduced IDO expression in the peripheral blood and induced sputum of children with allergic asthma compared to healthy children matched by age and gender, suggesting that IDO activity can inhibit airway allergic reactions and has a protective effect in the pathogenic mechanism of pediatric allergic asthma.

As mentioned in the introduction, plasma IDO activity and IL-10 levels were shown to be higher in allergy patients without clinical symptoms than in symptomatic allergy patients and healthy individuals [9]. Maneechotesuwan et al. [10] reported that the low IDO activity in the induced sputum of adult patients with mild intermittent and mild-to-moderate persistent asthma was enhanced by the inhalation of glucocorticoids, and this effect was possibly mediated by the stimulation of IL-10 production. Simvastatin also enhanced the regulatory effect of glucocorticoids on Th17/Treg imbalance by upregulating IDO and IL-10 expression and downregulating IL-6 and IL-23 expression [11]. Therefore, we speculate that IDO may play a crucial role in the mechanism underlying the imbalance of Th17/Treg differentiation, which may be related to cytokines such as IL-6 and IL-10. In addition, Bettelli et al. [22] found that Th17 and iTregs were generated from the same precursor, CD4+ Foxp3- T cells, which differentiated into Foxp3 + Tregs when treated with TGF-β and into CD4+ Foxp3- Th17 cells when treated with human TGF-β and IL-6, indicating that IL-6 is a key cytokine that determines whether CD4+ Foxp3− T cells convert to Tregs or Th17 cells. Interestingly, Baban et al. [23] reported an increased number of Tregs in the spleen of IL-6-knockout rats that were intravenously injected with the IDO inducer ISS-ODN, whereas subcutaneous injection of the IDO inhibitor 1-MT resulted in IL-6 expression in splenic pDCs, thereby leading to the conversion of Tregs to Th17 cells. The above findings further confirmed the role of IDO in the Th17/Treg differentiation imbalance as a molecular switch for the conversion of Th17 cells to Tregs under certain conditions. Consistent with the results from the studies mentioned above, the present clinical study revealed higher IDO levels and larger Treg populations in healthy children than in pediatric patients with allergic asthma, with a significant positive correlation between IDO level and Tregs; in contrast, IDO level exhibited a negative correlation with the Th17/Treg ratio, as patients with allergic asthma had markedly reduced IL-6 levels and significantly elevated IL-10 levels. Therefore, we speculate that IDO is involved in the differentiation of Th17/Tregs and may play a protective role by inhibiting IL-6 expression and enhancing IL-10 production, thereby promoting the conversion of Th17 cells to Tregs.

There were a few limitations of this study. For instance, the number of subjects enrolled in the study was relatively small due to difficulties in clinical specimen collection. The study aimed to evaluate children with asthma who were over 5 years old, and it did not adequately examine the characteristics of preschool children with symptoms of wheezing or panting. As a clinical investigation, this study did not conduct an in-depth investigation of the mechanism of action underlying the promotion of Th17 cell conversion to Tregs by IDO, and further validation with in vitro studies is needed.

## Conclusions

IDO activity exhibited a clear correlation with Th17/Treg differentiation in children with allergic asthma. We speculate that IDO upregulates Treg levels, possibly by stimulating IL-10 production and inhibiting IL-6 expression. Tt needs to be confirmed by further experiments IDO is likely a molecular switch for the conversion of Th17 cells to Tregs and potentially plays a protective role in the occurrence of asthma.

## Data Availability

The date and materials could be availabed from authors.

## Abbreviations

Treg: regulatory T cells
IDO: indoleamine 2,3-dioxygenase
DCs: dendritic cells
APCs: antigen-presenting cells
pDCs: plasmacytoid DCs
CTLA-4: T lymphocyte-associated antigen 4
TGF-β: transforming growth factor-β

## References

[1] Wilson RH, Whitehead GS, Nakano H, Free ME, Kolls JK, Cook DN (2009). Allergic sensitization through the airway primes Th17-dependent neutrophilia and airway hyperresponsiveness. Am J Respir Crit Care Med.

[2] Choy DF, Hart KM, Borthwick LA, Shikotra A, Nagarkar DR, Siddiqui S (2015). TH2 and TH17 inflammatory pathways are reciprocally regulated in asthma. Sci Transl Med..

[3] Zhao J, Lloyd CM, Noble A (2013). Th17 responses in chronic allergic airway inflammation abrogate regulatory T-cell-mediated tolerance and contribute to airway remodeling. Mucosal Immunol.

[4] Burchell JT, Strickland DH, Stumbles PA (2010). The role of dendritic cells and regulatory T cells in the regulation of allergic asthma. Pharmacol Ther.

[5] Hayashi T, Beck L, Rossetto C, Gong X, Takikawa O, Takabayashi K (2004). Inhibition of experimental asthma by indoleamine 2,3-dioxygenase. J Clin Invest..

[6] Gostner JM, Becker K, Kofler H, Strasser B, Fuchs D (2016). Tryptophan metabolism in allergic disorders. Int Arch Allergy Immunol.

[7] von Bubnoff D, Bieber T (2012). The indoleamine 2,3-dioxygenase (IDO) pathway controls allergy. Allergy.

[8] Raitala A, Karjalainen J, Oja SS, Kosunen TU, Hurme M (2006). Indoleamine 2,3-dioxygenase (IDO) activity is lower in atopic than in non-atopic individuals and is enhanced by environmental factors protecting from atopy. Mol Immunol.

[9] von Bubnoff D, Fimmers R, Bogdanow M, Matz H, Koch S, Bieber T (2004). Asymptomatic atopy is associated with increased indoleamine 2,3-dioxygenase activity and interleukin-10 production during seasonal allergen exposure. Clin Exp Allergy.

[10] Maneechotesuwan K, Supawita S, Kasetsinsombat K, Wongkajornsilp A, Barnes PJ (2008). Sputum indoleamine-2, 3-dioxygenase activity is increased in asthmatic airways by using inhaled corticosteroids. J Allergy Clin Immunol..

[11] Maneechotesuwan K, Kasetsinsombat K, Wamanuttajinda V, Wongkajornsilp A, Barnes PJ (2013). Statins enhance the effects of corticosteroids on the balance between regulatory T cells and Th17 cells. Clin Exp Allergy.

[12] Maneechotesuwan K, Ekjiratrakul W, Kasetsinsombat K, Wongkajornsilp A, Barnes PJ (2010). Statins enhance the anti-inflammatory effects of inhaled corticosteroids in asthmatic patients through increased induction of indoleamine 2, 3-dioxygenase. J Allergy Clin Immunol..

[13] Cho KS, Park MK, Kang SA, Park HY, Hong SL, Park HK (2014). Adipose-derived stem cells ameliorate allergic airway inflammation by inducing regulatory T cells in a mouse model of asthma. Mediators Inflamm.

[14] Albano GD, Di Sano C, Bonanno A, Riccobono L, Gagliardo R, Chanez P (2013). Th17 immunity in children with allergic asthma and rhinitis: a pharmacological approach. PLoS ONE.

[15] Wei B, Zhang H, Li L, Li M, Shang Y (2011). T helper 17 cells and regulatory T-cell imbalance in paediatric patients with asthma. J Int Med Res.

[16] Sakaguchi S, Yamaguchi T, Nomura T, Ono M (2008). Regulatory T cells and immune tolerance. Cell.

[17] Palomares O, Yaman G, Azkur AK, Akkoc T, Akdis M, Akdis CA (2010). Role of Treg in immune regulation of allergic diseases. Eur J Immunol.

[18] Durrant DM, Metzger DW (2010). Emerging roles of T helper subsets in the pathogenesis of asthma. Immunol Invest.

[19] Jalili RB, Forouzandeh F, Bahar MA, Ghahary A (2007). The immunoregulatory function of indoleamine 2, 3 dioxygenase and its application in allotransplantation. Iran J Allergy Asthma Immunol..

[20] Curti A, Trabanelli S, Salvestrini V, Baccarani M, Lemoli RM (2009). The role of indoleamine 2,3-dioxygenase in the induction of immune tolerance: focus on hematology. Blood.

[21] Puccetti P, Grohmann U (2007). IDO and regulatory T cells: a role for reverse signalling and non-canonical NF-kappaB activation. Nat Rev Immunol.

[22] Bettelli E, Carrier Y, Gao W, Korn T, Strom TB, Oukka M (2006). Reciprocal developmental pathways for the generation of pathogenic effector TH17 and regulatory T cells. Nature.

[23] Baban B, Chandler PR, Sharma MD, Pihkala J, Koni PA, Munn DH (2009). IDO activates regulatory T cells and blocks their conversion into Th17-like T cells. J Immunol..

